# Expression of Osteopontin in Patients with Thyroid Dysfunction

**DOI:** 10.1371/journal.pone.0056533

**Published:** 2013-02-20

**Authors:** Sara Reza, Asma Shaukat, Tariq M. Arain, Qasim Sarwar Riaz, Maria Mahmud

**Affiliations:** 1 Pathology Department, Quaid-e-Azam Medical College, Bahawalpur, Pakistan; 2 Department of Ophthalmology, Bahawal Victoria Hospital, Bahawalpur, Pakistan; 3 Department of Medicine, St. John Providence Health System, Warren, Michigan, United States of America; 4 Biology Department, Carleton University, Ottawa, Canada; Bambino Ges Children‚s Hospital, Italy

## Abstract

Thyroid dysfunctions are common endocrine problems. They are often misdiagnosed, misunderstood, and frequently overlooked. These disorders affect almost every aspect of health. Most of them remain undetected because the clinical assessment alone lacks both sensitivity and specificity. As it is not sufficient enough we require the biochemical tests to confirm the diagnosis. As a consequence there is still great interest in new biomarkers that complement existing diagnostic tools. Osteopontin, a glycoprotein that can be detected in plasma, was found to be upregulated in several patients with hyperthyroidism and downregulated in hypothyroid patients so it may represent a new biomarker. 100 patients with thyroid dysfunctions (50 hyperthyroid, 50 hypothyroid) and 100 normal subjects were included in the study. Osteopontin and other clinical parameters for diagnosis of thyroid disorders were measured. Osteopontin is positively correlated with T3 and T4 (r = 0.62 and r = 0.75 respectively) while it is negatively correlated with thyroid stimulating hormone (r = −0.52) showing a significant correlation (p-value <0.001). Our findings suggest that osteopontin might be useful as a novel prognostic biomarker in patients with impaired thyroid function.

## Introduction

Thyroid dysfunctions are the most common endocrine problems encountered in our clinical and endocrinology laboratory. Hyperthyroidism and hypothyroidism together are responsible for considerable morbidity in over 110 countries of the world among them most are developing countries [1]. The prevalence of these disorders in life time is approximately 5–10% [2]. According to estimation 27 million Americans [3], [4] have thyroid disease, and out of them about more than half remain undiagnosed [5]. Thyroid diseases are very often misdiagnosed, misunderstood, and frequently overlooked. They affect almost every aspect of health. Most of them remain undetected because the clinical assessment alone has less sensitivity and specificity and can suspect only up to 40% of symptomatic thyroid disorders. Only the biochemical tests can be used to confirm the diagnosis [6].

The function of normal thyroid gland is to control body metabolism, growth, development and maintain the internal environment. Thyroid is an important endocrine gland and produces two main hormones thyroxine (T4) and tri-iodo thyronine (T3) [7]–[9]. Both of these hormones are under control of thyroid stimulating hormone (TSH) released by anterior pituitary gland and in turn it is controlled by thyrotrophin releasing hormone (TRH) of hypothalamus. The spectrum of thyroid dysfunction ranges from hypothyroidism (under production) to hyperthyroidism (over production). Thyroid disorders may affect individuals belonging to any age and gender, but its occurrence is different in different geographical areas and in different age and sex groups. Therefore, various studies have been conducted to show the prevalence of thyroid disorders in Pakistani population. Akhtar et al (2002) reported that prevalence of hyperthyroidism and subclinical hyperthyroidism in all age groups was 5.1% and 5.8% respectively [1]. Hyperthyroidism and subclinical hyperthyroidism was found to be higher in females than males [1], [10]. Similarly, prevalence of hypothyroidism and subclinical hypothyroidism was 4.1% and 5.4% respectively and these disorders were also higher in females than males [1].

As mentioned earlier, most of the thyroid disorders remain undiagnosed as the clinical assessment alone is not sufficient enough to detect thyroid disorders so the biochemical tests are required to confirm the diagnosis [6]. As a consequence there is still great interest in new biomarkers that complement existing diagnostic tools and may facilitate risk stratification in patients with thyroid disease.

Osteopontin (OPN) is a molecule first identified in 1986 in osteoblasts and is known to be involved in the formation and calcification of bone [11]. The prefix of the word “osteo” indicates that the protein is expressed in bone and the suffix “-pontin” is derived from “pons,” the Latin word for bridge, and signifies osteopontin’s role as a linking protein. Osteopontin is a negatively charged phosphoglycoprotein which is composed of 300 amino acids and contain an arginine-glycine-aspartic acid cell binding sequence. It is located on the long arm of chromosome 4 region 13 (4q13) [12] and is found in different forms in the body i.e. full length osteopontin (OPN-FL), OPN-R elicited in various immune responses [13], [14], OPN-L with unknown function, intracellular OPN involved in migration, fusion and motility [15]–[18] and osteopontin-a, osteopontin-b and osteopontin-c, the cancer specific splice variants [19], [20]. It is expressed in fibroblasts [21], preosteoblasts, osteoblasts, osteocytes, odontoblasts, some bone marrow cells, hypertrophic chondrocytes, dendritic cells, macrophages [22] and T-cells, hepatocytes, smooth muscle, skeletal muscle, myoblasts [23], endothelial and epithelial cells [24], extraosseous cells in the inner ear, brain, kidney, deciduum, placenta and mammary glands. It is produced by local tissue cells and enters in the blood through attachment with CD44 and integrin (αvβ3) receptors [25], [26]. Pro‐inflammatory cytokines stimulate osteopontin gene transcription and expression [27]. Other mediators that can induce OPN upregulation include angiotensin II, transforming growth factor *β*(TGF*β*), tumor necrosis factor *α* (TNF*α*), interleukin‐1*β* (IL‐1*β*), nitric oxide (NO), hyperglycemia and hypoxia [28]–[31]. Osteopontin has important roles in normal physiological as well as pathological processes [32]. It is suggested that osteopontin plays a role in many diseases such as chronic inflammation, including Crohn’s disease [33], several types of cancer [34]–[36], autoimmune diseases [37], [38] i.e. Grave’s disease [39], obesity [40], [41], atherosclerosis [40]–[48] and cardiac fibrosis [40]. Osteopontin has multiple biological functions based on its structural modification and the environment in which it is expressed [12]. More information is required to understand the function of osteopontin in different disease states.

Keeping this in mind we investigated changes in serum oosteopontin with thyroid hormones and TSH levels induced by thyroid function disorders. In our study we aimed to demonstrate that serum osteopontin levels strongly correlate with clinical parameters of thyroid hormone dysfunction.

## Materials and Methods

### Study Design

This was a co relational study, carried out in Department of Pathology, Quaid-e-Azam Medical College, Bahawalpur from 1^st^ October 2010 to 30^th^ September 2011. The study was approved by ethics committee at Bahawal Victoria Hospital, Bahawalpur. Subjects fulfilling the required inclusion criteria were enrolled after a written and informed consent.

### Study Population

Subjects in our study were selected from the patients of age group 15–55 years, referred to Endocrinology lab unit (Department of Pathology, Quaid-e-Azam Medical College, Bahawalpur), for thyroid function tests with signs and symptoms suggestive of thyroid disorder. Brief clinical history and examination was done to rule out diabetes mellitus, renal disorder, liver disorder, or any other inflammatory or medical condition which would have influenced the parameters under study. No patient was taking any medication for their thyroid problem. Eye changes of patients were examined by ophthalmologist (Department of Ophthalmology, Bahawal Victoria Hospital, Bahawalpur), which included signs like upper eyelid retraction, edema, conjunctivitis, bulging eyes and visual acuity, visual field defects and optic disc to rule out other causes.

### Sample Collection

After noting the name, age and sex 6 ml of venous samples were drawn into an evacuated tube with aseptic precautions. Serum was separated by centrifugation at 3000 rpm for 15 minutes at 4°C. To avoid repetitive freeze and thaw cycles, different aliquots of one sample were generated, immediately frozen and stored at −80°C until analysis because osteopontin is highly sensitive to proteolytic degradation at higher temperatures and TSH assays are performed in batches.

### Sample Analysis

After screening by TSH levels, a total of 100 subjects (50 hyperthyroid and 50 hypothyroid) were selected from a larger cohort through simple random sampling and included in the study. As per serum TSH level the cases were further subdivided into two groups: Group I Hypothyroid with high TSH level than normal >4.2 mIU/L (since the upper limit of reference range for our laboratory is 4.2 mIU/L) and Group II Hyperthyroid with TSH level <0.4 mIU/L (as lower limit for reference range for our laboratory is 0.4 mIU/L).

After collection samples were further analyzed for fT3, fT4 and osteopontin. Thyroid function tests were done on Vitros ECi Immunodignostic System using kits from (Vitros ECi) while plasma osteopontin levels were determined with Enzyme-Linked Immunosorbent Assay by use of commercially available kit (ab100618 Osteopontin Human ELISA Kit by abcam) according to manufacturer’s instructions.

One hundred age and gender matched normal subjects were taken as a control and all the above parameters were also studied in these healthy subjects.

### Statistical Analysis

All data collected was subjected to standard statistical analysis, such as mean and standard error of mean for each of the parameters and expressed as mean±S.E. The statistical analysis was done by using SPSS version 16. Student’s t-test was used to compare the results of two groups for all parameters. Co-efficient of correlation and its significance (p-value) was calculated for osteopontin with TSH, T3 and T4. p-value less than 0.001 was considered statistically significant.

## Results

Patients were assigned different categories on the basis of clinical diagnosis which was made through history, examination; thyroid function tests and thyroid scan (Table 1). Grave’s disease was the most prevalent disorder in hyperthyroid group of patients while in case of hypothyroid group, iodine deficiency goiter was found to be the most common cause of hypothyroidism in our setting.

**Table 1:** Categories of patients on the basis of clinical diagnosis.

	HyperthyroidN = 50		HypothyroidN = 50	
	MaleN = 13	FemaleN = 37	MaleN = 8	FemaleN = 42
Grave’s disease	09 (69.2%)	32 (86.5%)	–	–
Toxic adenoma	–	02 (5.41%)	–	–
Toxic nodular goiter	04 (30.7%)	03 (8.11%)	–	–
Hashimoto thyroiditis	–	–	–	3 (7.14%)
Iodine deficiency goiter	–	–	8 (100%)	39 (92.8%)

Eye changes mentioned above were found in 23 patients of Grave’s disease out of which 19 were females and 4 were males.

Subject’s characteristics and anthropometric parameters are summarized in table 2. The common age group of having thyroid dysfunction was 30–40 years in our study population and the body mass index calculated for hypothyroid patients was more than the other two groups.

**Table 2:** Selected baseline characteristics for the study and control group.

		Patients		Controls
		Hyperthyroid	Hypothyroid	
Number		50	50	100
Sex	Females	37	42	77
	Males	13	8	24
Body mass index (kg/m^2^)		20.56±0.64	27.62±0.63	24.12±0.34
Age (x±S.E) (years)		34.92±1.55	33.3±1.46	31.23±0.92
Duration of disease		6 months-10 years	1–18 years	–

The hyperthyroid group of patients had significantly lower TSH and higher values of serum T3, T4 and osteopontin while hypothyroid group of patients had higher TSH and lower levels of serum T3, T4 and osteopontin than the healthy controls (Table 3).

**Table 3:** Measured parameters in patients with thyroid function disorders and healthy individuals.

Parameters	Group 1HyperthyroidN = 50mean±S.E	Group 2HypothyroidN = 50mean±S.E	Group 3Healthy controlsN = 100mean±S.E	p-valueby ANOVA
TSH (µIU/ml)	0.11±0.01	48.39±5.18	1.57±0.09	<0.001
f T3 (pmol/ml)	11.5±1.27	2.63±0.36	5.45±0.08	<0.001
f T4 (pmol/ml)	38.3±3.0	6.49±0.80	19.6±0.44	<0.001
OPN (ng/ml)	15.76±0.25	1.48±0.16	5.35±0.05	<0.001

TSH = Thyroid stimulating hormone, fT3 = Free tri-iodothyronine, fT4 = Free thyroxine, OPN = Osteopontin.

The coefficient correlation of serum osteopontin levels and typical clinical parameters of thyroid disorders were calculated. The results suggested that osteopontin is positively correlated with T3 and T4 while a strong negative correlation existed between osteopontin and thyroid stimulating hormone (Table 4).

**Table 4:** Correlation between OPN and TSH, fT3 and fT4 for all subjects.

Parameter	Pearson correlation (r)	p-value
OPN vs. TSH	−0.52	<0.001
OPN vs. fT3	0.62	<0.001
OPN vs. fT4	0.75	<0.001

TSH = Thyroid stimulating hormone, fT3 = Free tri-iodothyronine, fT4 = Free thyroxine, OPN = Osteopontin.

**Figure 1:**
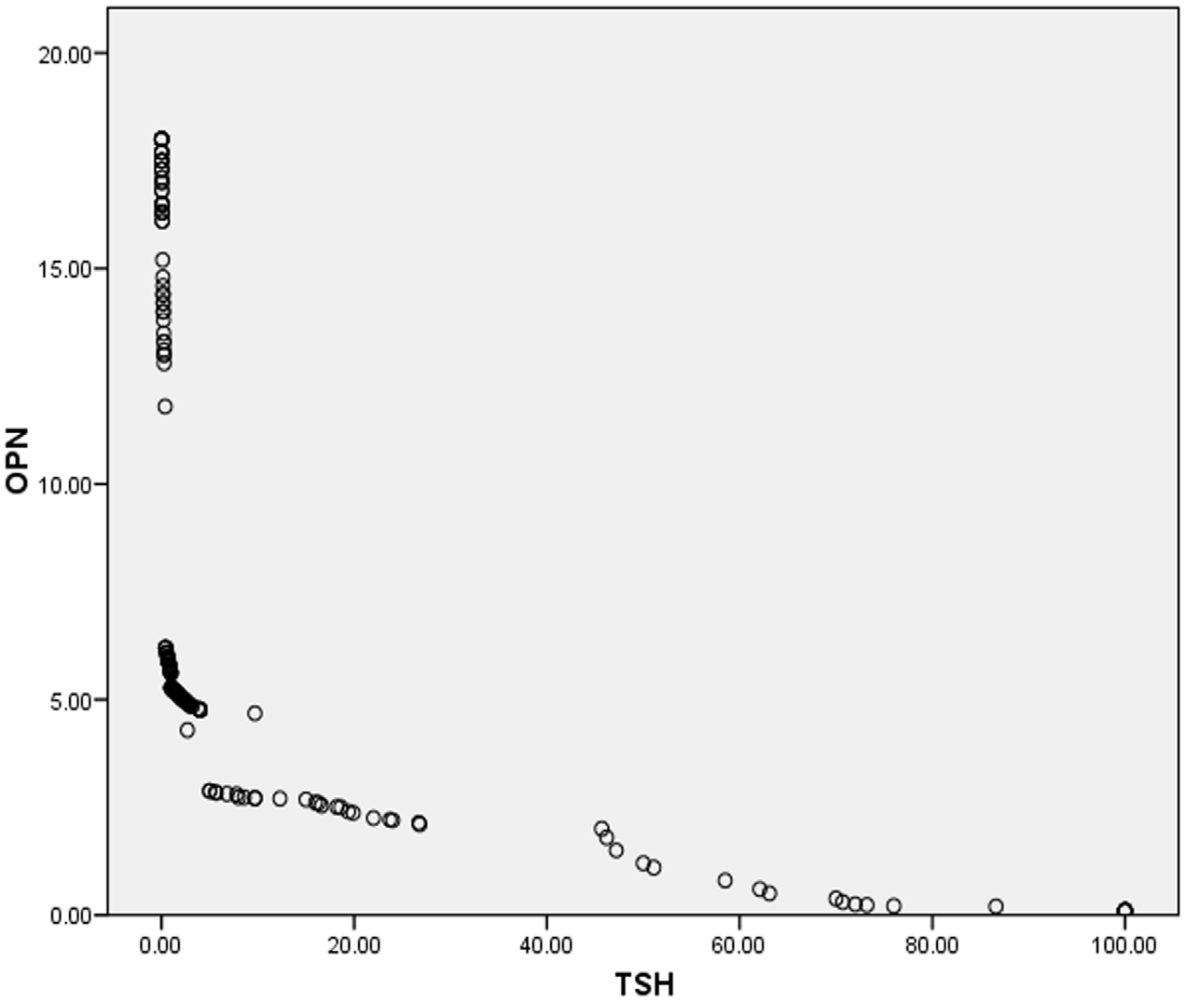
Figure **1. Negative correlation between osteopontin (OPN) and thyroid stimulating hormone (TSH).**

**Figure 2:**
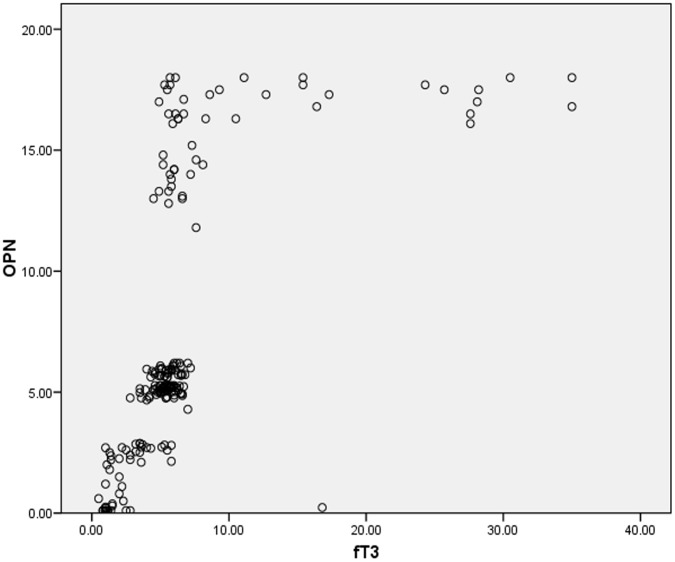
Figure **2. Positive correlation between osteopontin (OPN) and free triiodothyronine (fT3).**

**Figure 3:**
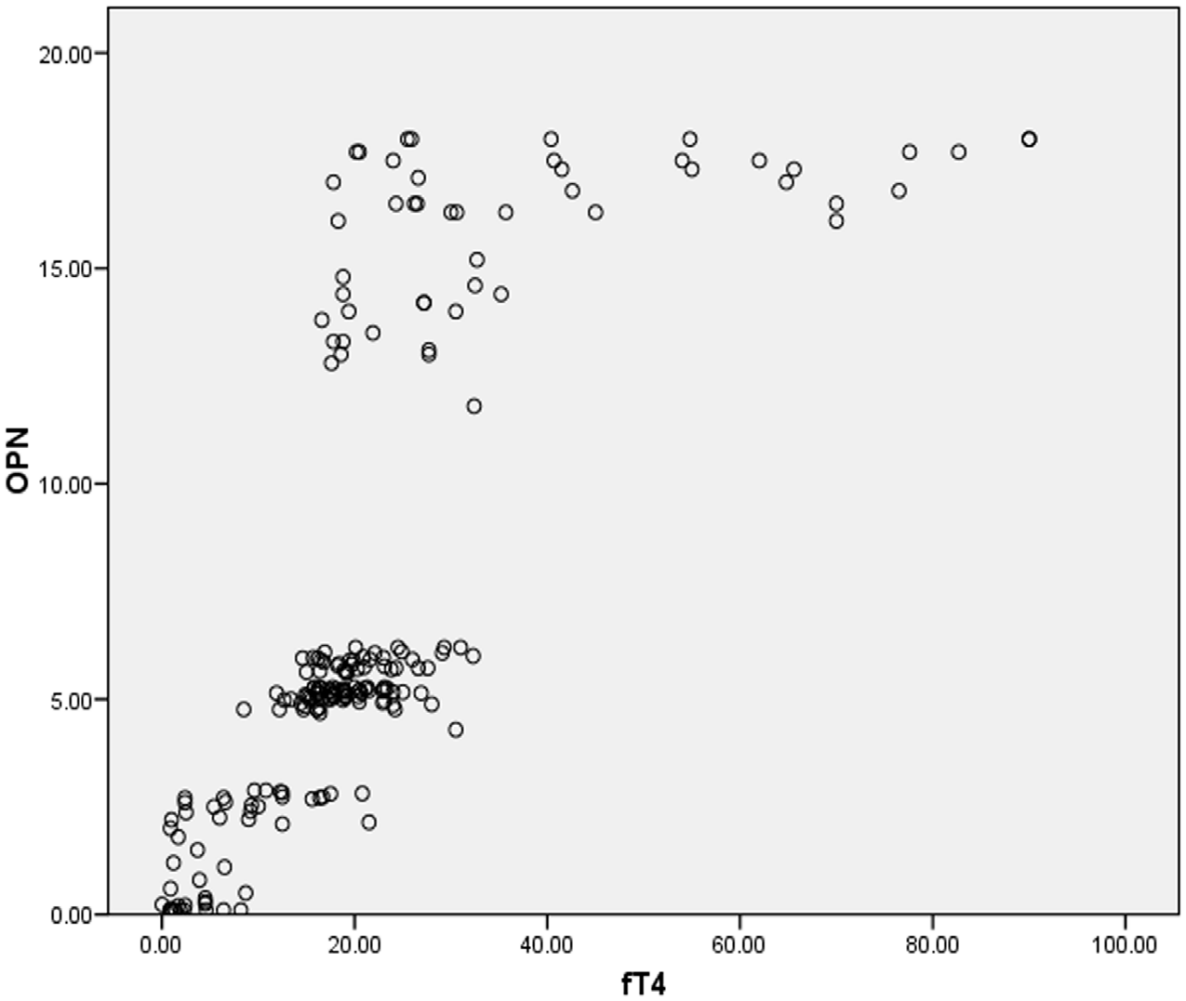
Figure **3. Positive correlation between osteopontin (OPN) and free thyroxine (fT4).**

Figures 1, 2 and 3 shows scatter plots for the correlation of osteopontin with thyroid stimulating hormone, free tri-iodo thyronine and free thyroxine for total 200 subjects included in our study indicating the negative correlation between osteopontin (OPN) and TSH; and the positive correlation of OPN with fT3 and fT4.

## Discussion

Among 50 hyperthyroid patients, 74% were females and 26% were males while in hypothyroid group there were 84% females and 16% males which showed that females are more prone to have thyroid disorders. Our findings are in accordance with that of Akhtar et al [1] and Mansoor et al [2] but the ratio of female to male was lower to the study done by Vanderpump et al [49] as it was a population based study and ours is a hospital based study so the findings cannot be generalized and epidemiological studies are necessary to provide authentic data about prevalence and sex distribution of thyroid dysfunction. These prevalence differences between subjects in this study and others may be due to differences in age, gender, family history and pathophysiologic conditions. Results of this study, however, are within the prevalence limits established by other studies. Also common age group of having thyroid dysfunction was 30–40 years in our study which is comparable to study by Baral et al in eastern Nepal [50].

The present study, to our knowledge, is the first to report pathological impact of osteopontin in adult population of Pakistan with thyroid dysfunction. As mentioned earlier, osteopontin (OPN) was not only found to be involved in the formation and calcification of bone, but also in processes like inflammation, cell adhesion and migration and prevention of apoptosis [51] because of its expression by various other tissues of the body. The pattern of change in serum osteopontin levels observed in our study i.e. elevated in hyperthyroidism and decreased in hypothyroidism, may be due to these various cell processes going on in the thyroid gland under the influence of osteopontin, yet the exact function of osteopontin in thyroid dysfunction has not been determined.

Our results are comparable with the study on patients with Grave’s disease in Chinese population [39]. According to this study in patients with Grave’s disease, serum OPN levels were elevated which coincided with an increase in OPN receptor coexpression and enhancement in proinflammatory cytokine and chemokine production. Significant difference observed in serum osteopontin levels between normal and hyperthyroid patients in this study was due to the fact that OPN promotes pathogenesis of autoimmune diseases by inducing immune cell activation and migration and inflammatory cytokine production. The correlation between TSH and OPN (r = −0.56) is slightly more than our study (r = −0.52) which is probably due to small sample size and different inclusion criteria. All hyperthyroid patients (TSH <0.4 mIU/l) were included in our study irrespective of the cause of over production of thyroid hormones.

Data of our study showed that majority of hyperthyroid patients (69.2%) were diagnosed as Grave’s disease but we cannot state with confidence that this is the only thyroid dysfunction responsible for deranged osteopontin levels because similar results were obtained in all other cases of hyperthyroidism i.e. toxic adenoma (5.41%) and toxic nodular goiter (14%). Also, all cases of hypothyroidism showed decreased levels of osteopontin. But the effect on serum osteopontin concentration is more marked in patients with hyperthyroidism. Large randomized controlled trials could provide more definitive evidence.

Another study analyzed animal model of hypothyroid mice created by ingestion of propylthiouracil (PTU). After PTU ingestion, the animals had significant decrease in thyroid hormones (T3 and T4) and assays revealed an increase of OPN mRNA expression in the aorta and heart. Hypothyroid animals treated with T3 showed a reduction in aortic OPN mRNA expression. This data in contrast to our data indicated that the increase of OPN mRNA and protein expression occurs in cardiovascular tissues of hypothyroid mice [52]. The reason may be that there exists a difference between expression of animal and human osteopontin. Noteworthy, osteopontin overexpression is associated with thyroid cancers in humans [53], [54] because osteopontin is a cytokine which regulates cell trafficking within the immune system and plays an important role in initiation, progression, and transplantation of malignant tumors [55].

### Conclusion

These findings suggest that alterations in thyroid status can change serum osteopontin concentration. So the measurement of this parameter may provide useful information regarding the diagnosis of thyroid disease.

Based on the results of this investigation, it should be considered that there is still little knowledge regarding the basic pathophysiology of correlation between osteopontin and thyroid dysfunction. Considerably more work will need to be done to determine whether osteopontin concentration is affected in all cases of thyroid dysfunction (especially in subclinical cases) and whether restoration of euthyroidism brings back serum osteopontin level to normal. More information on the effects of treatment would help us establish a greater degree of accuracy on this matter.
